# Hepatitis C Virus Nonstructural Protein 5A Inhibits MG132-Induced Apoptosis of Hepatocytes in Line with NF-κB-Nuclear Translocation

**DOI:** 10.1371/journal.pone.0131973

**Published:** 2015-07-02

**Authors:** Xia Jiang, Tatsuo Kanda, Shuang Wu, Shingo Nakamoto, Masato Nakamura, Reina Sasaki, Yuki Haga, Takaji Wakita, Hiroshi Shirasawa, Osamu Yokosuka

**Affiliations:** 1 Departments of Gastroenterology and Nephrology, Chiba University, Graduate School of Medicine, Chiba, Japan; 2 Departments of Molecular Virology, Chiba University, Graduate School of Medicine, Chiba, Japan; 3 Department of Virology II, National Institute of Infectious Diseases, Tokyo, Japan; Saint Louis University, UNITED STATES

## Abstract

**Background:**

Hepatitis C virus (HCV) infection is one of the major causes of cirrhosis and hepatocellular carcinoma. HCV nonstructural protein 5A (NS5A) is an attractive antiviral target and plays an important role in HCV replication as well as hepatocarcinogenesis. The aim of this study was to assess the effect of HCV NS5A protein in the abrogation of apoptotic cell death induced by the proteasome inhibitor MG132.

**Methods:**

Apoptotic responses to MG132 and the expression of molecules involved in NF-κB signaling pathways in human hepatocytes were investigated with or without the expression of HCV NS5A.

**Results:**

HCV NS5A protected HepG2 cells against MG132-induced apoptosis, in line with NF-κB-nuclear translocation. A similar NF-κB-nuclear translocation was observed in Huh7 cells infected with HCV JFH1. In agreement with this, after treatment with MG132, HCV NS5A could elevate the transcription of several NF-κB target genes such as BCL2 and BCLXL to inhibit MG132-induced apoptosis in hepatocytes. HCV HCV NS5A also enhanced phosphorylation of IκBα. Consistent with a conferred prosurvival advantage, HCV NS5A reduced MG132-induced poly(adenosine diphosphate-ribose) polymerase cleavage.

**Conclusions:**

HCV NS5A expression enhances phosphorylation of IκBα, liberates NF-κB for nuclear translocation and downregulates MG132-induced apoptotic pathways in human hepatocytes. It is possible that the disruption of proteasome-associated apoptosis plays a role in the pathogenesis of HCV infection.

## Introduction

Hepatitis C virus (HCV) is one of the major risk factors for hepatocellular carcinoma (HCC) [1,2]. HCV is a global health issue, with more than 185 million individuals chronically infected worldwide [3]. Although HCV drug treatment regimens have depended on HCV genotypes [4], these regimens have been improved, and multiple direct-acting antivirals (DAAs) therapies will soon become globally realistic options [3].

HCV has a positive-strand RNA genome of approximately 9.6 kb, which encodes at least 4 structural (core, E1, E2 and p7) and 6 nonstructural (NS2, NS3, NS4A, NS4B, NS5A and NS5B) proteins. At least 4 of the 10 proteins, namely core, NS3, NS5A and NS5B, play roles in several potentially oncogenic pathways [5]. The HCV NS5A protein is a ~447 amino acid phosphoprotein with an N-terminal amphipathic alpha helix and 3 structural domains [6, 7]. HCV NS5A is important for HCV replication [8] and is involved in multiple cellular processes [9, 10]. HCV NS5A has been shown to block hepatic apoptosis mediated by tumor necrosis factor (TNF) [11, 12], lipopolysaccharide (LPS) [13] or thapsigargin [14], suggesting that the disruption of this apoptosis may play a role in the pathogenesis of HCV infection.

The proteasome inhibitor MG132 has been shown to induce apoptosis in HepG2 cells [15, 16]. MG132 activates c-Jun N-terminal kinase (JNK), which initiates apoptosis and also inhibits NF-κB activation [17]. MG132 dramatically sensitizes HDAC-inhibitor-mediated apoptosis, at least partly, through the ER stress response [18]. HCV NS5A modulates JNK and activates NF-κB [19, 20]. Alterations in cell survival contribute to the pathogenesis of human liver diseases and viral carcinogenesis [21, 22]. In the present study, we examined the effect of HCV NS5A protein in abrogating apoptotic cell death induced by MG132. Our results demonstrate that HCV NS5A protein suppresses MG132-mediated apoptosis.

## Methods

### Plasmids, Cells and Virus

Plasmid pCXN2 was generously provided by Prof. J. Miyazaki (Osaka University) [23]. pCXN2-HCV NS5A vectors were described previously [14]. pCXN2-NS5A plasmids with the HCV genotype 1b NS5A, including 2 wide-type, 2 intermediate-type, and 1 mutant-type interferon sensitivity determining region (ISDR, aa2209-aa2248) were named pCXN2-NS5A-W1 and pCXN2-NS5A-W2, pCXN2-NS5A-I1 and pCXN2-NS5A-I2, and pCXN2-NS5A-M1, respectively [14]. The human hepatoma cell HepG2 was cultured in Dulbecco’s modified Eagle’s medium (Invitrogen, Carlsbad, CA, USA) containing 10% heat-inactivated fetal bovine serum, 100 units/ml penicillin and 100 μg/ml streptomycin (Sigma, St. Louis, MO, USA) under 5% CO2 at 37°C. Stable HepG2 cell lines as control cells, which expressed pCXN2 [14], and HepG2-NS5A cells, which expressed various HCV genotype 1b NS5A proteins, have been described previously [13, 14]. Huh7 cells were infected with JFH1 genotype 2a as described elsewhere [24, 25].

### Treatment of Cells with MG132

HepG2 control or HepG2-NS5A cells were placed in 6-well plates and incubated with 0–10 μM MG132 (Z-Leu-Leu-Leu-al) (Sigma-Aldrich, St. Louis, MO, USA). After 24–48 hours of incubation, cells were fixed for 30 minutes with methanol, washed 3 times with water, stained for 30 minutes with 0.1% crystal violet, and air-dried [13].

### Reporter Assays for NF-κB Activity

HepG2 cells were seeded onto a 6-well plate and 24 hours later co-transfected with 0.2 μg reporter plasmid pNFκB-luc (PathDetect Cis-Reporting Systems; Agilent Technologies, Santa Clara, CA, USA) and 0.2 μg pCXN2-HCV NS5A vector or pCXN2 control vector using Effectene transfection reagents (Qiagen, Hilden, Germany). Forty-eight hours after transfection, cells were harvested using reporter lysis buffer (Toyo Ink, Tokyo, Japan), and luciferase activities were determined with a luminometer (Luminescencer-JNR II AB-2300, ATTO, Tokyo, Japan).

### RNA Purification, Real-time RT-PCR and Human NF-κB Signaling Targets PCR Array

Cellular RNA was extracted using the RNeasy Mini Kit (Qiagen). One microgram of RNA was reverse-transcribed with a PrimeScript RT^2^ First Strand Kit (Qiagen). PCR was performed on cDNA templates using primers specific for B-cell CLL/lymphoma (BCL2), BCL2-like 1 (BCL2L1/BCL-X/BCLXL) and glyceraldehyde-3-phosphate dehydrogenase (GAPDH), which were purchased from Qiagen. A Human NF-κB Signaling Targets Real-time RT-PCR Array was performed according to the manufacturer's protocol. The data were analyzed by PCR Array Data Analysis Software (http://www.sabiosciences.com/pcrarraydataanalysis.php).

### Western Blot Analysis

Cells were harvested with sodium dodecyl sulfate sample buffer. After sonication, cell lysates were subjected to electrophoresis on 5–20% polyacrylamide gels and transferred onto polyvinylidene difluoride membranes (ATTO). Membranes were probed with specific antibodies for poly(adenosine diphosphate-ribose) polymerase (PARP), nuclear factor of kappa light polypeptide gene enhancer in B cells inhibitor, alpha (IκBα), phosphorylated IκBα (Ser32) (Cell Signaling Technology, Danvers, MA, USA) and GAPDH (Santa Cruz Biotechnology, Santa Cruz, CA, USA). After washing, membranes were incubated with secondary horse-radish peroxidase-conjugated antibodies. Signals were detected by means of enhanced chemiluminescence (GE Healthcare Japan, Tokyo, Japan) and scanned with an image analyzer LAS-4000 and Image Gauge (version 3.1) (Fuji Film, Tokyo, Japan). Band intensities were determined by ImageJ software [26].

### Transfection and Apoptosis Assay

Approximately 1 x 10^5^ HepG2 cells were placed on 6-well tissue culture plates (Iwaki Glass, Tokyo, Japan) 24 hours prior to transfection. Cells were transfected with 0.3 μg of pCXN2 or pCXN2-NS5A vector using Effectene transfection reagents (Qiagen) according to the manufacturer’s protocol. After 48 hours of transfection, cells were treated with 0–10 μM MG132 (Sigma-Aldrich) for 24 hours, and APOPercentage Apoptosis Assay (Biocolor, Belfast, Northern Ireland) was used to quantify apoptosis according to the manufacturer’s instructions. The transfer and exposure of phosphatidylserine to the exterior surface of the membrane has been linked to the onset of apoptosis. Phosphatidylserine transmembrane movement results in the uptake of APOPercentage dye by apoptotic cells. Purple-red stained cells were identified as apoptotic cells by light microscopy. The number of purple-red cells per 300 cells was counted as previously described [14].

### Measurement of Caspase-3/-7 Activities

The caspase-Glo 3/7 assay (Promega, Madison, WI, USA) was used to detect caspase-3 and caspase-7 activities according to the manufacturer’s instructions [14].

### Immunofluorescence Study

Cells were washed and fixed with 3.7% formaldehyde, followed by blocking with 3% horse serum albumin. Cells were incubated with an NF-κB P65 (D14E12) antibody (Cell Signaling) for 16 hours at 4°C. Cells were washed and incubated with anti-rabbit immunoglobulin secondary antibody conjugated with Alexa Fluor 555 (Cell Signaling) for 1 hour at room temperature. Nuclear staining was performed with Hoechst 33342, trihydrochloride, trihydrate (Molecular Probes, Eugene, OR, USA). Finally, cells were washed and mounted for confocal microscopy (ECLIPSE TE 2000-U, Nikon, Tokyo, Japan), and the images were superimposed digitally to allow for fine comparisons.

### Statistical Analysis

Results were expressed as mean ± standard deviation (SD). Statistical analyses were performed using Student’s t-test. A P-value of < 0.05 was considered statistically significant. All statistical analyses were performed using DA Stats software (O. Nagata, Nifty Serve: PAF01644).

## Results

### MG132 reduced cell viabilities in HepG2 control cells more than in HepG2-NS5A cells

**Fig 1:**
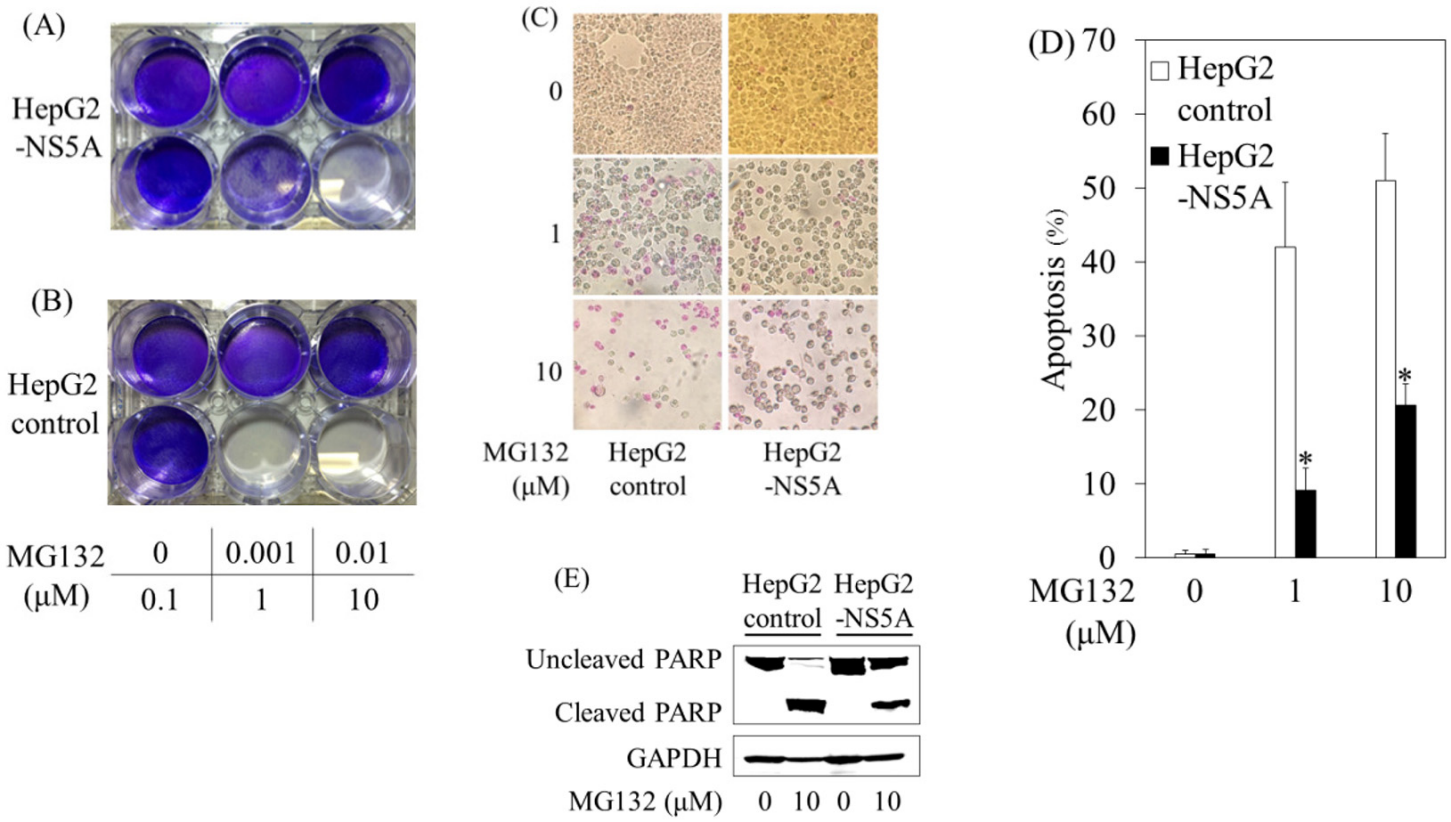
HCV NS5A reduced MG132-induced apoptosis. HepG2-NS5A (*A*) and HepG2 control (*B*) cells were treated with 0–10 μM MG132 for 48 hours, and stained with 0.1% crystal violet. *C*, *D*, HepG2 control or HepG2-NS5A cells were treated with 0–10 μM MG132 for 24 hours, and apoptosis was quantified with the APOPercentage Apoptosis Assay according to the manufacturer’s instructions. *C*, Purple-red-stained cells were identified as apoptotic cells using light microscopy (40X). *D*, The number of purple-red cells per 300 cells was counted as previously described [14]. *P<0.05, compared to HepG2 control cells by Student's t-test. *E*, Western blot analyses of poly(adenosine diphosphate-ribose) polymerase (PARP) and glyceraldehyde-3-phosphate dehydrogenase (GAPDH) expression in HepG2 control or HepG2-NS5A cells at 24 hours after treatment with or without 10 μM MG132.

HepG2 cells stably transfected with HCV NS5A (HepG2-NS5A) were used to examine its effects on MG132-mediated cell death. We treated HepG2-NS5A and HepG2 control cells with MG132 at various concentrations and analyzed cell death 48 hours later (Fig 1A and 1B). Compared with HepG2-NS5A cells, a much higher level of cell death was observed in HepG2 control cells treated with 10 μM MG132.

### HCV NS5A reduced MG132-induced apoptosis

The quantification of apoptosis showed a significant 4.6-fold or 2.5-fold increase in apoptosis in HepG2 cells treated with 1 μM or 10 μM MG132 compared with HepG2-NS5A cells treated with 1μM or 10 μM MG132, respectively (Fig 1C and 1D). We also investigated whether HCV NS5A interfered with apoptosis, using western blot analysis, to detect PARP cleavage, which is thought to be one of the hallmarks of apoptosis. Whereas PARP of HepG2-NS5A cells was expressed at higher levels, cleaved PARP induced by 10 μM MG132 was observed more strongly in HepG2 control cells than in HepG2-NS5A cells (Fig 1E). Caspase-3/-7 activities measured in the presence of MG132 were increased in HepG2 control cells (~1.5-fold) compared with HepG2-NS5A cells. These results showed that HCV NS5A protected the human hepatoma cell line HepG2 from MG132-induced apoptosis.

### HCV NS5A enhanced MG132-induced NF-κB-nuclear translocation

**Fig 2:**
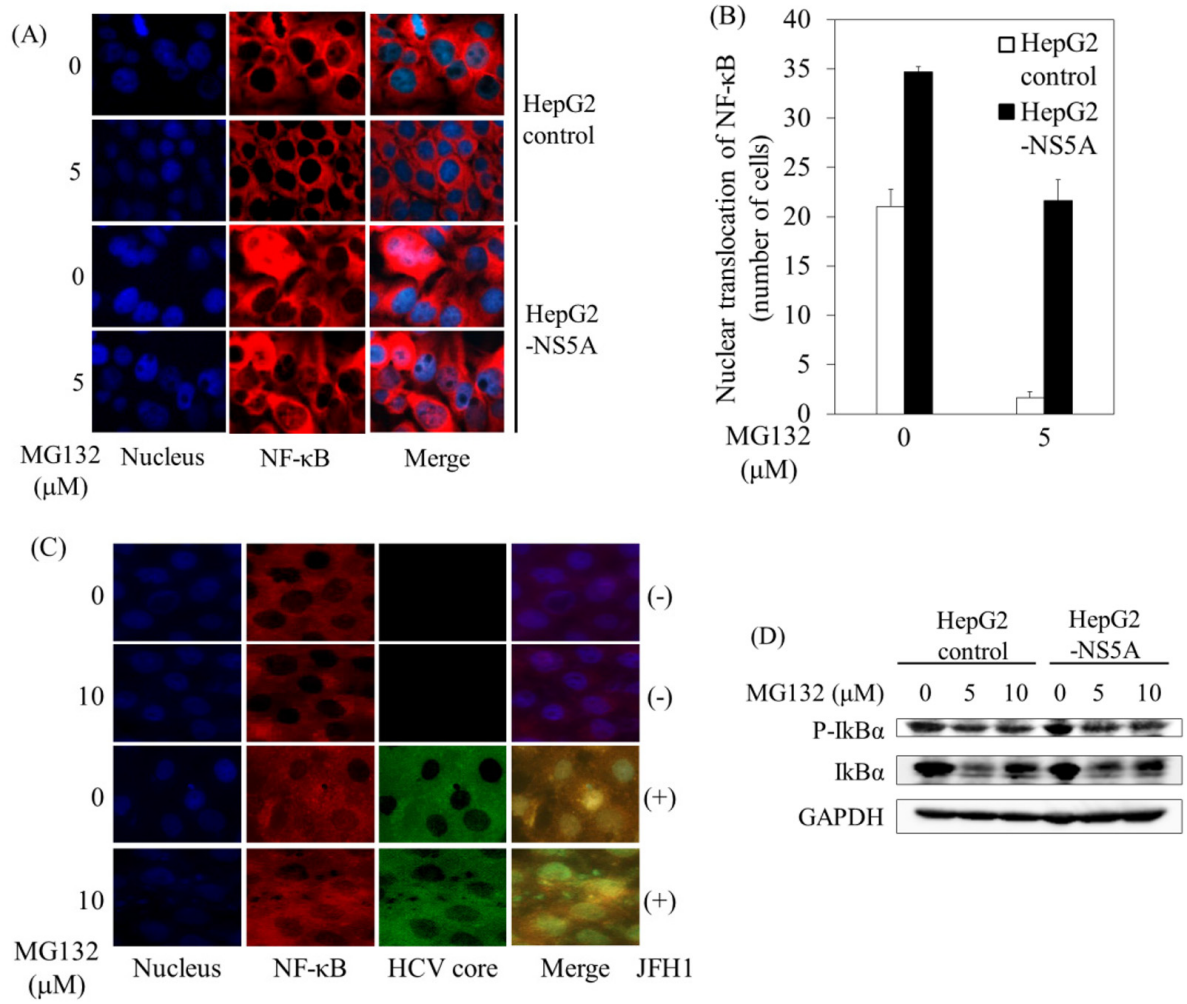
HCV NS5A enhanced MG132-induced NF-κB p65 nuclear translocation. *A*, HepG2 control and HepG2-NS5Acells were cultured for 6 hours with or without 5 μM MG132. Confocal microscope high-power view demonstrates that NF-κB p65 nuclear localization was detected (x200). Nuclear staining was performed with Hoechst 33342, trihydrochloride, trihydrate (blue). Localization of NF-κB p65 was detected with anti-NF-κB p65 primary antibody, and Alexa-Fluor-555 secondary antibody (red). Merge images of A and B were superimposed digitally to allow for fine comparisons. NF-κB p65 nuclear translocation was observed (pink). *B*, The number of NF-κB p65 nuclear-translocated cells per field was counted at low-power view (40X). *C*, Huh7 cells infected with or without HCV JFH1 genotype 2 strain were incubated for 6 hours with or without 5 μM MG132. Nuclear staining was performed with Hoechst 33342, trihydrochloride, trihydrate (blue). NF-κB p65 nuclear localization was detected with anti-NF-κB p65 primary antibody and anti-rabbit Alexa-Fluor-555 secondary antibody (red). HCV was detected using anti-HCV core primary antibody and an Alexa-Fluor-488 anti-mouse secondary antibody (green). *D*, HCV NS5A expression enhances phosphorylation of IκBα. Western blot analyses of phosphorylated IκBα (Ser32) (P-IκBα), IκBα and glyceraldehyde-3-phosphate dehydrogenase (GAPDH) expression in HepG2 control or HepG2-NS5A cells at 8 hours after treatment with or without MG132.

MG132, a compound that can inhibit the activation of NF-κB, initiates apoptosis [17, 27]. To further analyze the underlying mechanisms of this process, we compared the localization of NF-κB p65 in HepG2 control cells with that in HepG2-NS5A cells in the presence or absence of 5 μM MG132 using immunofluorescence (Fig 2A and 2B). After 24 hours, cells were stained with rabbit monoclonal NF-κB p65 antibody. NF-κB nuclear localization was more readily detected in HepG2-NS5A cells than in HepG2 control cells in the presence and absence of 5 μM MG132 (Fig 2A and 2B). In accordance with these results, NF-κB-nuclear translocation was observed in Huh7 infected with HCV JFH1 (Fig 2C). A reporter assay also revealed that NF-κB transcriptional activity measured in the presence of MG132 was increased in HepG2-NS5A cells (~4.1-fold) compared with HepG2 control cells. We treated both cell lines, HepG2 control and HepG2-NS5A with 0, 5, and 10 μM MG132 for 8 hours, collected samples and subjected them to SDS-PAGE and western blotting by antibody specific for phosphorylated IκBα, IκBα or GAPDH (Fig 2D). Band intensity rates of phosphorylated IκBα/total IκBα were 1.0-, 1.19- and 0.87-fold in HepG2 control, and they were 1.41-, 1.33-, and 1.18-fold in HepG2-NS5A cells. Together, our data demonstrated that HCV NS5A may enhance NF-κB activation, resulting in reduced MG132-induced apoptosis in hepatocytes.

### Different amino acid sequences of ISDR have no impact on MG132-induced apoptosis

**Fig 3:**
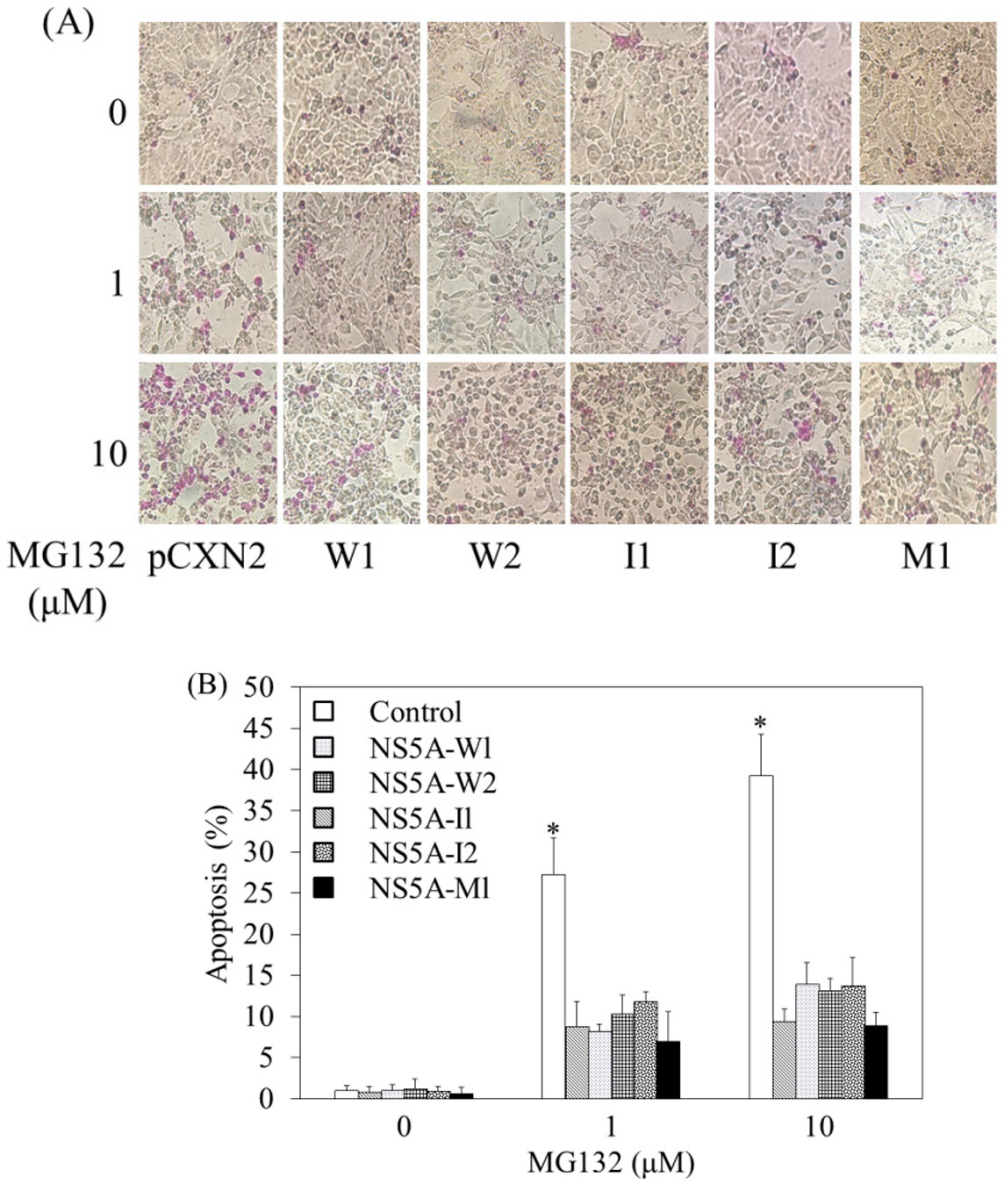
HCV NS5A interferon-sensitivity determining region (ISDR) has no impact on MG132-induced apoptosis. *A*, *B*, HepG2 cells were transfected with 0.3 μg of each vector as indicated. After 24 hours, cells were treated with 0–10 μM MG132 for 24 hours, and apoptosis was evaluated by the APOPercentage Apoptosis Assay. *A*, Purple-red-stained cells were identified as apoptotic cells by light microscopy (40X). *B*, The number of purple-red cells is shown. *P<0.05, compared to HepG2 control cells without MG132 treatment by Student's t-test.

HCV NS5A ISDR has an association with the treatment response in interferon-including regimens against chronic HCV infection [28]. As we reported previously [14], we made HCV NS5A expression vectors, having 0 (W1 and W2), 1 (I1) or 2 (I2), or 5 amino acid changes (M1) in the HCV NS5A2209-2248 region, compared with those of HCV-J (wild type). We examined MG132-induced apoptosis after transient transfection of each HCV NS5A expression plasmid into HepG2 cells. After 24 hours, cells were treated with MG132 for 24 hours and apoptosis was evaluated as previously described (Fig 3A and 3B). HCV NS5A containing different sequences in the ISDR region have a similar effect on MG132-induced apoptosis as compared to wild type sequence. It may be possible that NS5A ISDR interacts with host protein and blocks MG132-induced apoptosis.

### NF-κB-associated genes upregulated in HepG2-NS5A cells

To obtain further mechanistic insights into HCV NS5A for NF-κB-signaling pathways, we used a pathway-specific gene array to identify HCV NS5A target genes in HepG2-NS5A cells by comparison with HepG2 control cells. We extracted total RNAs from both cell lines for studying the influence of HCV NS5A on NF-κB-signaling pathway-associated gene expression using real-time PCR arrays (Tables 1 and 2). Out of 84 NF-κB-signaling pathway-associated genes, 6 (7.1%) genes [chemokine (C-C motif) ligand 5 (CCL5/RANTES), chemokine (C-C motif) ligand 2 (CCL2/MCP-1), synaptosomal-associated protein, 25 kDa (SNAP25), colony stimulating factor 1 (macrophage) (CSF1), chemokine (C-X-C motif) ligand 10 (CXCL10/IP-10) and complement factor B (CFB)] were significantly upregulated by 1.5-fold or greater in HepG2-NS5A cells compared with HepG2 control cells (n = 3, p <0.05) (Table 1). Of interest, chemokine genes, such as CCL5, CCL2 and CXCL10, could activate human hepatic stellate cells, which are associated with hepatic fibrosis [29–31], and CXCL10 is an important gene for the treatment response of interferon treatment [32]. CCL5, CCL2 and CXCL10 have anti-apoptotic functions [33–35]. CSF1 and SNAP25 were associated with the development and differentiation of myeloid cells and the nervous system, respectively. CFB is classified as one of the innate immune response genes.

**Table 1:** NF-κB-signaling pathway-associated genes significantly upregulated by more than 1.5-fold in HepG2-NS5A cells compared with HepG2 control cells.

Symbol	Description	Function	Fold change	p-value
CCL5	Chemokine (C-C motif) ligand 5	Anti-apoptotic gene	4.67	0.00005
CCL2	Chemokine (C-C motif) ligand 2	Anti-apoptotic gene	3.4998	0.0004
SNAP25	Synaptosomal-associated protein, 25 kDa	Development and differentiation	3.3894	0.00002
CSF1	Colony stimulating factor 1 (macrophage)	Development and differentiation	3.1068	0.032
CXCL10	Chemokine (C-X-C motif) ligand 10	Anti-apoptotic gene	2.6657	0.0049
CFB	Complement factor B	Innate immune response gene	2.0428	0.0003

Fold-change is proportional to the ratios of gene expression levels between HepG2-NS5A and HepG2 control cells. Positive numbers indicate that the genes were upregulated in HepG2-NS5A cells. Each experiment was performed at least 3 times independently.

**Table 2:** NF-κB-signaling pathway-associated genes significantly downregulated by more than 1.5-fold in HepG2-NS5A cells compared with HepG2 control cells.

Symbol	Description	Function	Fold change	p-value
TRAF2	TNF receptor-associated factor 2	Apoptosis-related gene	-1.54	0.00029
BCL2L1	BCL2-like 1	Anti-apoptotic gene	-1.63	0.00038
CXCL2	Chemokine (C-X-C motif) ligand 2	Inflammatory chemokine	-1.72	0.0044
IL1A	Interleukin 1, alpha	Inflammatory cytokine	-1.74	0.039
IL2RA	Interleukin 2 receptor, alpha	Apoptosis-related gene	-1.79	0.0499
CD83	CD83 molecule	Innate immune response gene	-1.85	0.003
CDKN1A	Cyclin-dependent kinase inhibitor 1A (p21, Cip1)	Type I interferon-response gene	-1.88	0.00003
C3	Complement component 3	Inflammatory-related gene	-1.89	0.000004
CCND1	Cyclin D1	Stress response gene	-1.95	0.00007
BIRC3	Baculoviral IAP repeat containing 3	Anti-apoptotic gene	-2.00	0.000007
TNFRSF1B	Tumor necrosis factor receptor superfamily, member 1B	Apoptosis-related gene	-2.11	0.0086
RELB	V-rel reticuloendotheliosis viral oncogene homolog B	NF-κB pathway	-2.33	0.00017
PTGS2	Prostaglandin-endoperoxide synthase 2 (prostaglandin G/H synthase and cyclooxygenase)	Apoptosis-related gene	-2.87	0.000001
PDGFB	Platelet-derived growth factor beta polypeptide	Stress response gene	-3.07	0.0007
F3	Coagulation factor III (thromboplastin, tissue factor)	Inflammatory-related gene	-4.41	0.000001
CCL22	Chemokine (C-C motif) ligand 22	Inflammatory chemokine	-6.62	0.00097
LTB	Lymphotoxin beta (TNF superfamily, member 3)	Inflammatory cytokine	-12.47	0.0027
IL8	Interleukin 8	Inflammatory cytokine	-16.42	0.00068
TNF	Tumor necrosis factor	Inflammatory cytokine	-109.94	0.0040

Fold-change is proportional to the ratios of gene expression levels between HepG2-NS5A and HepG2 control cells. Negative numbers indicate that the genes were downregulated in HepG2-NS5A cells. Each experiment was performed at least 3 times independently.

### NF-κB-associated genes downregulated in HepG2-NS5A cells

On the other hand, out of 84 NF-κB-signaling pathway-associated genes, 19 genes were significantly downregulated by 1.5-fold or greater in HepG2-NS5A cells compared with HepG2 control cells (n = 3, p <0.05) (Table 2). Inflammatory cytokines and chemokines such as tumor necrosis factor (TNF), interleukin 8 (IL8), lymphotoxin beta (TNF superfamily, member 3) (LTB), chemokine (C-C motif) ligand 22 (CCL22), interleukin 1, alpha (IL1A) and chemokine (C-X-C motif) ligand 2 (CXCL2) were suppressed. Inflammatory-related genes such as coagulation factor III (thromboplastin, tissue factor) (F3), tumor necrosis factor receptor superfamily, member 1B (TNFRSF1B), complement component 3 (C3), and interleukin 2 receptor alpha (IL2RA) were also suppressed. Apoptosis-related genes such as prostaglandin-endoperoxide synthase 2 (prostaglandin G/H synthase and cyclooxygenase) (PTGS2/COX-2), TNFRSF1B, IL2RA and TNF receptor-associated factor 2 (TRAF2) were suppressed, although anti-apoptotic genes such as BCLXL and baculoviral IAP repeat containing 3 (BIRC3/c-IAP1) were also suppressed. Type I interferon-response gene, cyclin-dependent kinase inhibitor 1A (p21, Cip1) (CDKN1A), and innate immune response gene, CD83, were also inhibited. Stress response genes such as platelet-derived growth factor beta polypeptide (PDGFB) and cyclin D1 (CCND1) were also suppressed. In the NF-κB pathway, transcription factor V-rel reticuloendotheliosis viral oncogene homolog B (RELB) was suppressed (Table 2).

Although HCV NS5A had an impact on several NF-κB-associated genes in hepatocytes, we also found that the expression of BCL2 mRNA and BCLXL mRNA were up-regulated approximately 1.46-fold and 1.98-fold, respectively, in HepG2-NS5A cells, compared to HepG2 control cells after 8 hours of treatment with 10 μM MG132. These results also suggested that HCV NS5A protein could elevate the transcription of several NF-κB target genes, such as BCL2 and BCLXL, to inhibit MG132-induced apoptosis in hepatocytes.

## Discussion

In the present study, the MG132-induced apoptotic pathway is inhibited in HCV NS5A-expressing HepG2 cells by activation of NF-κB. HCV NS5A influences NF-κB-targeting gene expression and blocks PARP cleavage and apoptosis induced by MG132, leading to hepatocyte survival. The present study supported the previous observation that MG132 reduced the viability of HepG2 cells in a time- and dose-dependent manner and that the effect was in tight connection with the induction of apoptosis [15]. Emanuele et al. [15] also reported that MG132 caused the degradation of PARP and that protease inhibitors could have potential as a treatment against HCC.

HCV NS5A has anti-apoptotic properties [9–14]. Our results (Fig 2D) supported the previous observation that HCV NS5A could activate NF-κB via phosphorylation of IκBα [36]. The hyperphosphorylated form of IκBα is targeted for degradation, releasing p50/p65 (or Rel) complex to translocate to the nucleus [37]. HCV NS5A also controls the interferon (IFN)-induced double-stranded RNA (dsRNA)-activated protein kinase, PKR [38], and PKR as a eukaryotic initiation factor 2, alpha subunit (eIF2a) kinase, regulates the NF-κB, p38MAPK and insulin pathways [39]. In the present study, we observed that HCV NS5A enhanced NF-κB-nuclear translocation in the presence or absence of MG132 (Fig 2). Although the PKR-binding region of HCV NS5A includes ISDR [38], HCV NS5A containing different sequences in the ISDR region have a similar effect on MG132-induced apoptosis as compared to wild type sequence (Fig 3). It may be possible that NS5A ISDR interacts with host protein and blocks MG132-induced apoptosis.

NF-κB activation was blocked by either adenovirus-mediated overexpression of Iκ0042α suppressor or pretreatment with MG132 in lung cancer cells [40]. NF-κB activation confers resistance to TNF-mediated apoptosis in hepatocytes, as TNF is one of the important cytokines for eradication of HCV from the liver [41]. HCV NS5A could play a role in the activation of NF-κB. In the present study, TNF expression was also suppressed in HepG2-NS5A cells, compared with HepG2 control cells (Table 2). These results are in agreement with the previous reports of HCV NS5A-transgenic animals [41]. NF-κB is also critical for the apoptosis of HCC and plays a role in the sensitivity to sorafenib [42]. Further studies will be needed for this issue.

Intracellular protein degradation is an important mechanism, which includes ubiquitin-proteasome and ubiquitin-independent proteasome pathways for the modulation of certain proteins and the elimination of damaged proteins. MG132 effectively blocks the proteolytic activity of the 26S proteasome complex [43]. It is evident that several viruses are able to manipulate the ubiquitin-proteasome pathway by redirecting the cellular ubiquitin machinery to enable replication. Growing evidence suggests that ubiquitin-proteasome and ubiquitin-independent proteasome pathways are also involved in controlling the stability of HCV proteins such as core [43, 44], p7 [45], NS2 [46] and NS5A [47]. Of interest, it has been reported that, in the presence of HCV, STAT1 and STAT3 proteins were ubiquitinated and that the degradation was blocked by MG132 [48, 49]. HCV may inhibit interferon responses via proteasomal degradation of JAK/STAT pathway components [49]. It is possible that HCV NS5A may interact with intracellular protein degradation pathways [47]. Although proteasome inhibition elevates IκBα levels and leads to inhibition of NF-κB activity [50,51], we observed that HCV NS5A expression enhances phosphorylation of IκBα and liberates NF-κB for nuclear translocation in the present study (Fig 2D).

In conclusion, HCV NS5A enhances the phosphorylation of IκBα, which in turn enhances NF-κB-nuclear translocation and down-regulates MG132-induced apoptotic pathways in human hepatocytes. Together, the disruption of proteasome-associated apoptosis may play a role in the pathogenesis of HCV infection.
