# Correction: Hepatitis C Virus Nonstructural Protein 5A Inhibits MG132-Induced Apoptosis of Hepatocytes in Line with NF-κB-Nuclear Translocation

**DOI:** 10.1371/journal.pone.0134068

**Published:** 2015-07-21

**Authors:** The PLOS ONE Staff

There is an error in the first sentence of the third paragraph in the Discussion. The correct sentence is: NF-κB activation was blocked by either adenovirus-mediated overexpression of IκBα suppressor or pretreatment with MG132 in lung cancer cells [40]. The publisher apologizes for this error.
